# Physio-Morphological, Biochemical and Transcriptomic Analyses Provide Insights Into Drought Stress Responses in *Mesona chinensis* Benth

**DOI:** 10.3389/fpls.2022.809723

**Published:** 2022-02-10

**Authors:** Danfeng Tang, Changqian Quan, Yang Lin, Kunhua Wei, Shuangshuang Qin, Ying Liang, Fan Wei, Jianhua Miao

**Affiliations:** ^1^Guangxi Key Laboratory of Medicinal Resources Protection and Genetic Improvement, Guangxi Botanical Garden of Medicinal Plants, Nanning, China; ^2^Guangxi Engineering Research Center of TCM Resource Intelligent Creation, Guangxi Botanical Garden of Medicinal Plants, Nanning, China

**Keywords:** *Mesona chinensis* Benth, biochemical analyses, transcriptome, plant hormone signal transduction, brassinosteroid biosynthesis, transcription factor

## Abstract

Drought stress affects the normal growth and development of *Mesona chinensis* Benth (MCB), which is an important medicinal and edible plant in China. To investigate the physiological and molecular mechanisms of drought resistance in MCB, different concentrations of polyethylene glycol 6000 (PEG6000) (0, 5, 10, and 15%) were used to simulate drought conditions in this study. Results showed that the growth of MCB was significantly limited under drought stress conditions. Drought stress induced the increases in the contents of Chla, Chlb, Chla + b, soluble protein, soluble sugar, and soluble pectin and the activities of superoxide dismutase (SOD), catalase (CAT), total antioxidant capacity (TAC), hydrogen peroxide (H_2_O_2_), and malondialdehyde (MDA). Transcriptome analysis revealed 3,494 differentially expressed genes (DEGs) (1,961 up-regulated and 1,533 down-regulated) between the control and 15% PEG6000 treatments. These DEGs were identified to be involved in the 10 metabolic pathways, including “plant hormone signal transduction,” “brassinosteroid biosynthesis,” “plant–pathogen interaction,” “MAPK signaling pathway-plant,” “starch and sucrose metabolism,” “pentose and glucuronate interconversions,” “phenylpropanoid biosynthesis,” “galactose metabolism,” “monoterpenoid biosynthesis,” and “ribosome.” In addition, transcription factors (TFs) analysis showed 8 out of 204 TFs, *TRINITY_DN3232_c0_g1* [*ABA-responsive element* (*ABRE*)*-binding transcription factor1, AREB1*], *TRINITY_DN4161_c0_g1* (*auxin response factor, ARF*), *TRINITY_DN3183_c0_g2* (*abscisic acid-insensitive 5-like protein, ABI5*), *TRINITY_DN28414_c0_g2* (*ethylene-responsive transcription factor ERF1b, ERF1b*), *TRINITY_DN9557_c0_g1* (*phytochrome-interacting factor, PIF3*), *TRINITY_DN11435_c1_g1, TRINITY_DN2608_c0_g1*, and *TRINITY_DN6742_c0_g1*, were closely related to the “plant hormone signal transduction” pathway. Taken together, it was inferred that these pathways and TFs might play important roles in response to drought stress in MCB. The current study provided important information for MCB drought resistance breeding in the future.

## Introduction

Drought is a complex and natural phenomenon mainly caused by less precipitation in a certain period, which has the characteristics of frequency, intensity, and duration (Mishra and Singh, 2010). Drought stress is one of the most harmful abiotic stresses and can adversely affect the growth and development of crops (Fathi and Tari, 2016; Fahad et al., 2017; Hussain et al., 2018; Khan et al., 2020). In the agricultural production areas, especially in southern China, due to the changes of climate and environment and the development of the economy in recent decades, severer and more frequent drought periods seriously hinder plant growth and development resulting in a large number of agricultural production losses (Tang et al., 2019).

Water is one of the important environmental conditions for crop growth and development, which has a significant impact on crop morphology, physiological metabolism, yield, and quality. Drought stress affects the water relations of plants at the cellular, tissue, and organ levels causing physical damage, physiological and biochemical disruptions, and molecular changes (Beck et al., 2007) like photosynthesis, respiration, antioxidant, hormonal metabolism, and gene expression (Bhargava and Sawant, 2013). Under drought stress, the relative water content, chlorophyll content, and plant biomass decrease significantly in two wheat cultivars (Ma et al., 2012). Plants can reduce osmotic potential through the accumulation of osmotic substances (Bray, 1993), such as proline, soluble sugar, betaine, organic acid, free amino acid, and other organic substances (Wang et al., 2010) to maintain the normal water demand and promote drought tolerance. Drought stress can disturb the self-regulation balance of cells, lead to the excessive accumulation of reactive oxygen species (ROS), cause membrane peroxidation, changes in membrane fluidity and ion transport, damage of biological macromolecules, destruction of chloroplast structure, metabolic disorder, and finally result in plant death (Gill and Tuteja, 2010). In the process of long-term evolution, to maintain the steady-state balance of ROS in cells, plant cells have formed a complex antioxidant defense system, which includes superoxide dismutase (SOD), peroxidase (POD), catalase (CAT), ascorbic acid peroxidase (APX), and so on (Apel and Hirt, 2004; Jaleel et al., 2009). Plant hormones can be used as signal molecules to mediate the drought stress response of plants. Usually, plant hormones will react together to cope with drought stress, such as inhibiting the synthesis of growth and developmental hormones like auxin (IAA), gibberellin (GA), zeatin nucleoside (ZR), and promoting the synthesis of growth inhibitory hormones like abscisic acid (ABA) and jasmonic acid (JA) (Lu, 2020), etc. Among these, the plant hormone ABA plays a central role. ABA, as a long-distance signal, mediates the response of the whole plant to drought stress, and also as a cellular signal, regulates the expression of drought stress response genes (Zhang et al., 2006). Transcription factors (TFs) are the main regulators that control gene clusters. A single TF, called regulon, can regulate the expression of many target genes by the specific binding of the TF to *cis*-acting element in the promoters of the target genes; Dehydration-responsive element binding protein 1 (DREB1)/C-repeat binding factor (CBF) (DREB1/CBF) and DREB2 regulons function in ABA-independent gene expression, while the ABA-responsive element (ABRE) binding protein (AREB)/ABRE binding factor (ABF) (AREB/ABF) regulon functions in ABA-dependent gene expression (Nakashima et al., 2009). In addition, TFs such as *AP2/ERF*, *MYB*, *MYC*, *NAC*, and *WRKY* are also involved in abiotic stress-responsive gene expression (Zhou et al., 2020).

*Mesona chinensis* Benth (MCB) is one of the important medicinal plants in tropical and subtropical areas, including southern China and Southeast Asian countries (Tang et al., 2020). MCB contains polysaccharides, flavonoids, triterpenoids, phenols, and other chemical components (Lin et al., 2013) with multiple biological activities, such as antioxidation (Yen and Hung, 2000), antihypertensive effects (Yeha et al., 2009), hypolipidemic effects (Li et al., 2010), antibiosis (Liu and Feng, 2008), etc. MCB can grow in slope, forest, ditch, and stream or dry sandy land and possesses strong environmental adaptability, however, it is not resistant to drought and cold (Zhao et al., 2011). If the water shortage is serious in summer and the temperature is too low in winter, the MCB plants will grow poorly or even die, resulting in yield reduction (Su et al., 2008). In recent years, due to high temperature and less rainfall in autumn and winter in Guangxi, moderate drought often occurs and lasts for about 3 months (Ren et al., 2017), which seriously affects the sustainable development of the MCB industry. Therefore, analysis of physiological, biochemical, and molecular responses of MCB seedlings under drought stress is of great significance for the study of drought resistance of MCB under the background of global climate change.

Polyethylene glycol (PEG) can hinder the plant conducting tissues and simulation of drought stress by PEG induces drought stress on the plants (Jiang et al., 1995). PEG molecules with a molecular weight greater than 3000 are hard to enter the cell wall space (Rubinstein and Turner, 1982) and are not apparently absorbed (Tarkow et al., 1996). PEG induces significant water stress in plants and is non-toxic (Emmerich and Hardegree, 1990). PEG6000 is mainly used to determine the drought stress-related information of plants (Turkan et al., 2005). Studies have reported that PEG6000 is used to simulate drought in many medicinal plants, such as *Astragalus membranaceus* (Liang et al., 2021), *Sophora alopecuroides* (Yan et al., 2020), *Fagopyrum tataricum* (Huang et al., 2021), *Sophora moorcroftiana* (Li et al., 2015), etc. However, there are fewer reports on the mechanism of drought stress in *Mesona Chinensis* Benth. In this study, PEG6000 was used to simulate drought in an experimental room. To study the mechanism of drought resistance, different PEG6000 concentrations (0, 5, 10, and 15%, named as PEG0, PEG5, PEG10, and PEG15, respectively) were utilized and the morphological, physiological, biochemical, and molecular changes were characterized and determined. The current study was of great significance for the study of drought resistance of MCB and provided important information for the improvement of drought tolerance in this plant.

## Materials and Methods

### Plant Materials and Treatments

The cutting seedlings of MCB were used in this study. The cuttings were planted in germination boxes using small white stones and treated individually with 5, 10, and 15% PEG6000 (W/V) solutions. The different concentrations (5, 10, and 15%) of PEG6000 were prepared in 0.5× Hoagland solution and the 0.5× Hoagland solution was used as control (CK). In total, there were four treatments (CK, PEG5, PEG10, and PEG15) in this study, and each contained three replicates. All the materials were cultured under about 2400 LUX red light intensity and at 26 ± 1°C for 16/8 h (light/dark) periods. The seedlings were cultured with tap water for 3 days to make the MCB plants adapt to the environment, then different levels of PEG6000 solutions were used for drought treatments. The different concentrations of PEG6000 solutions were changed every 2 days. After 8 days of drought stress, at least three plantlets were sampled for data collection of agronomic traits, meanwhile, the 3rd–5th apical leaves were harvested for biochemical determination and RNA sequencing. The samples were immediately frozen with liquid nitrogen and stored at −80°C for further analysis.

### Biochemical Determinations

Three plants were harvested for measurements of the fresh weight of the whole plant, root, and above-ground, plant height, and the stem diameter, then we took the average values. The Chla and Chlb concentration was measured by using the method of Gao (2000). The soluble sugar (SS) content was determined by the anthrone colorimetry method (Buysse and Merckx, 1993) and the soluble protein (SP) content was measured by Coomassie brilliant blue G-250 staining method (Bradford, 1976). The SOD activity was determined as described by Giannopolitis and Ries (1977). The POD activity was determined according to the reference (Hemeda and Klein, 1990). The CAT activity estimation was performed according to Goldblith and Proctor (1950). Malondialdehyde (MDA) content was estimated according to the method of Kramer et al. (1991). Hydrogen peroxide (H_2_O_2_) content was detected as described by Velikova et al. (2000). All the data were collected with three replicates.

### RNA Extraction, Library Preparation, and Illumina Sequencing

The 3rd–5th apical leaves of seedlings from the control (CK) and 15% PEG6000 treatments were used for RNA isolation by RNA extraction Kit (TRIzol Reagent, Invitrogen, Carlsbad, CA, United States). Then the RNA integrity and quality were assessed. We used the Illumina TruSeq™ RNA sample preparation Kit (San Diego, CA, United States) to prepare RNA-seq transcriptome libraries. Then the RNAseq libraries were sequenced in a single lane on an Illumina Hiseq x ten/NovaSeq 6000 sequencer (Illumina, San Diego, CA, United States) for 2 × 150 bp paired-end reads.

### *De novo* Assembly and Annotation

The raw paired-end reads were trimmed and quality controlled by Sickle^1^ and SeqPrep^2^ with default parameters. Then the clean data were utilized to perform *de novo* assembly with Trinity^3^ (Grabherr et al., 2011). All the assembled transcripts were searched against the NCBI protein non-redundant (NR), Cluster of Orthologous Groups of proteins (COG), and Kyoto Encyclopedia of Genes and Genomes (KEGG) databases using BLASTX to identify the proteins that had the highest sequence similarity with the given transcripts and a typical cut-off *E*-values < 1.0 × 10^––5^ was set. BLAST2GO^4^ (Conesa et al., 2005) was employed to get Gene Ontology (GO) annotations of unique assembled transcripts for describing molecular functions (MF), biological processes (BP), and cellular components (CC). We used the KEGG (Goto and Kanehisa, 2000) to conduct metabolic pathway analysis. The RNA-seq data were deposited in Sequence Read Archive (SRA) database with accession number PRJNA777790.

### Differential Expression Analysis and Functional Enrichment

To identify differential expression genes (DEGs) between the samples of control and 15% PEG6000 (CK vs. 15% PEG), we calculated the expression level of each transcript according to the transcripts per million reads (TPM) method and quantified gene abundances with RSEM (Li and Dewey, 2011). Differential expression analysis was performed using the DESeq2 (Love et al., 2014) with *Q*-value ≤ 0.05, DEGs with | log2FC| > 1 and *Q*-value ≤ 0.05 were considered as significantly differentially expressed genes. Moreover, functional-enrichment analyses including KEGG and GO were carried out to identify DEGs, which were significantly enriched in GO terms and metabolic pathways at Bonferroni-corrected *P*-value ≤ 0.05 in comparison with the whole-transcriptome background. GO functional enrichment and KEGG pathway analysis were performed by Goatools and KOBAS (Xie et al., 2011).

### Quantitative Real-Time PCR Analysis

We randomly selected 12 differentially expressed unigenes between PEG0 and PEG15 samples for qRT-PCR analysis. The qRT-PCR primers were listed in Supplementary Table 1. The qRT-PCR was conducted by using ChamQ CYBR qPCR Master Mix (Vazyme, China) and QuantStudio 7 Flex system (Applied Biosystems, ABI, Waltham, MA, United States). The RNA and cDNA used in qRT-PCR were the same as those used for RNA sequencing. *GAPDH* was employed as an internal control and the qPCR system and condition were according to the reference (Tang et al., 2021). Three biological repeats were used for each.

### Statistics

SPSS (Version.17, SPSS Inc., Chicago, IL, United States) software was used for one-way variance analysis (ANOVA). The data means were analyzed using the Duncan test for statistical significance (*P*-values ≤ 0.05).

## Results

### Drought Stress Strongly Limited the Growth and Development of *Mesona chinensis* Benth

**FIGURE 1:**
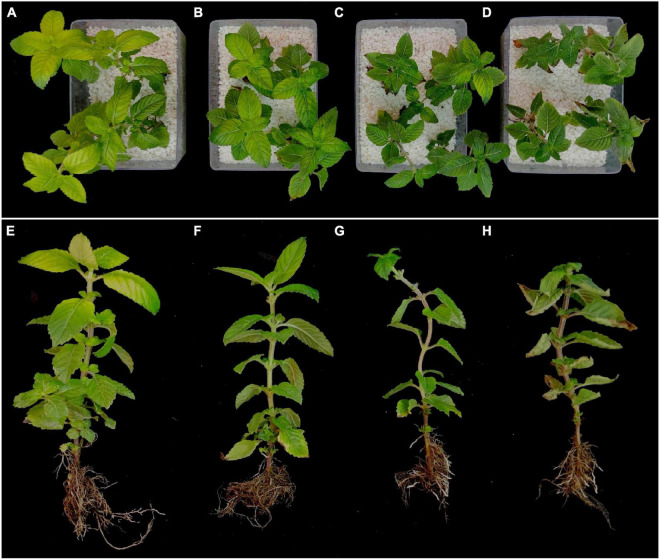
Phenotypic changes of *Mesona Chinensis* Benth (MCB) under normal (CK) and drought stress conditions. **(A,E)** The plants under normal condition. **(B,F)** The plants under 5% PEG6000 condition. **(C,G)** The plants under 10% PEG6000 condition. **(D,H)** The plants under 15% PEG6000 condition.

**FIGURE 2:**
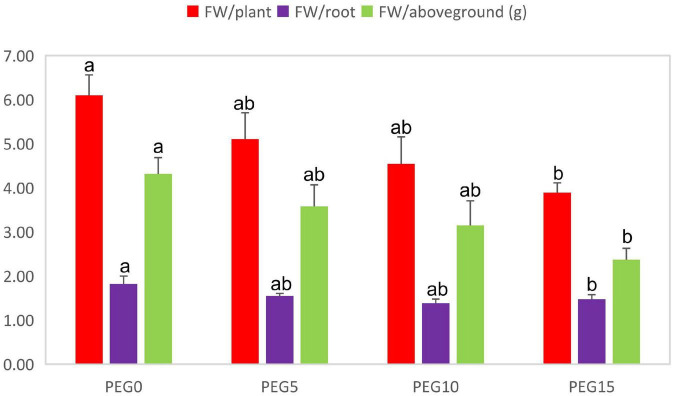
Changes in the fresh weight of the whole plant, root, and above-ground under different drought stress conditions. Different letters indicate the significant difference at the 0.05 level.

**FIGURE 3:**
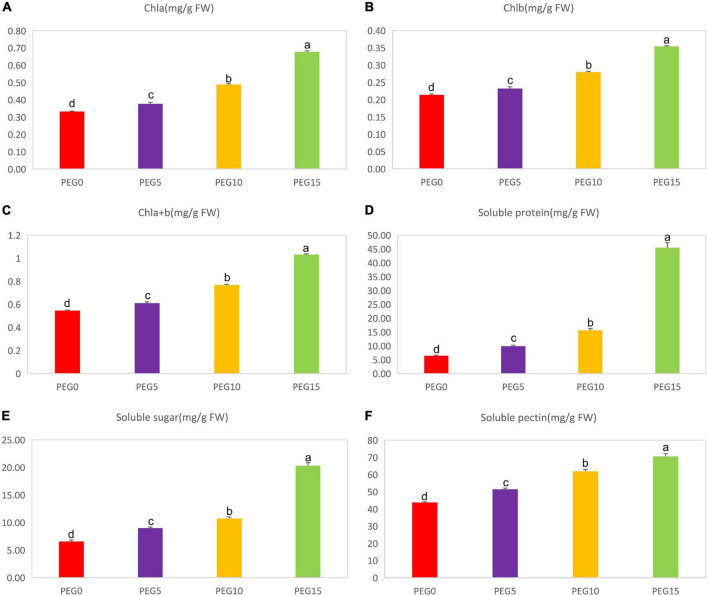
Changes of the contents of Chla, Chlb, Chla + b, soluble protein, soluble sugar, and soluble pectin under different drought stress conditions. **(A–F)** The contents of Chla, Chlb, Chla + b, soluble protein, soluble sugar, and soluble pectin under different drought stress conditions, respectively. Different letters indicate the significant difference at the 0.05 level.

After 8 days of drought stress, the growth of MCB was significantly limited (Figure 1). With the increase of PEG6000 concentrations, the fresh weight of the whole plant, root, and above-ground significantly decreased (Figure 2). Compared to the control, the fresh weight of the whole plant, root, and above-ground was reduced by 36.19, 19.00, and 45.03% under 5, 10, and 15% PEG6000 conditions, respectively. The contents of Chla, Chlb, Chla + b, soluble protein, soluble sugar, and soluble pectin were significantly increased with the increasing concentration of PEG6000 (Figure 3), which were positively correlated to PEG6000 concentration. Under 15% PEG6000 condition, the contents of Chla, Chlb, Chla + b, soluble protein, soluble sugar, and soluble pectin were increased by 103.95, 65.26, 88.80, 608.16, 209.47, and 60.89%, respectively, in comparison with the control.

### Drought Stress Promoted Antioxidant Enzyme Activities of *Mesona chinensis* Benth

**FIGURE 4:**
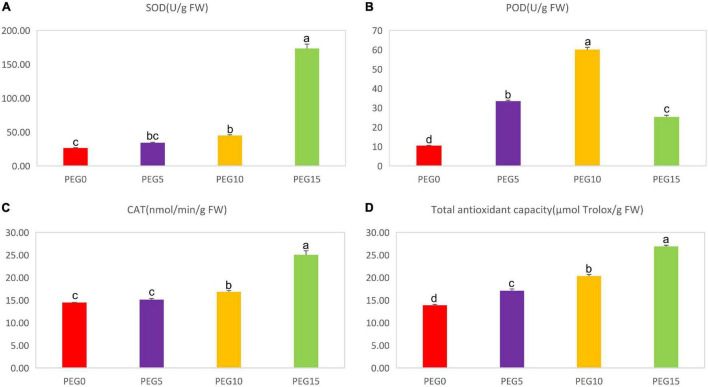
Changes of the antioxidant enzyme activities under different drought stress conditions. **(A–D)** The contents of superoxide dismutase (SOD), peroxidase (POD), catalase (CAT), and total antioxidant capacity (TAC) under different drought stress conditions, respectively. Different letters indicate the significant difference at the 0.05 level.

To better understand the possible mechanisms of the physiological response of MCB to drought stress, the activities of the antioxidant enzymes (SOD, POD, and CAT) and the total antioxidant capacity (TAC) were monitored under different concentrations of PEG6000. As shown in Figure 4, the SOD, POD, and CAT activities and the TAC were significantly affected in various drought treatments. The SOD, CAT, and TAC were increased with the increasing concentration of PEG6000. Under 15% PEG6000 concentration, they were increased by 559.66, 73.56, and 94.18%, respectively, compared to the control. However, the POD activity was increased then decreased with the increasing concentration of PEG6000 (Figure 4B). Compared to the control, the POD activities in PEG5, PEG10, PEG15 treatments were increased by 220.32, 475.91, and 142.16%, respectively.

### Drought Stress Improved Malondialdehyde and Hydrogen Peroxide Contents of *Mesona chinensis* Benth

**FIGURE 5:**
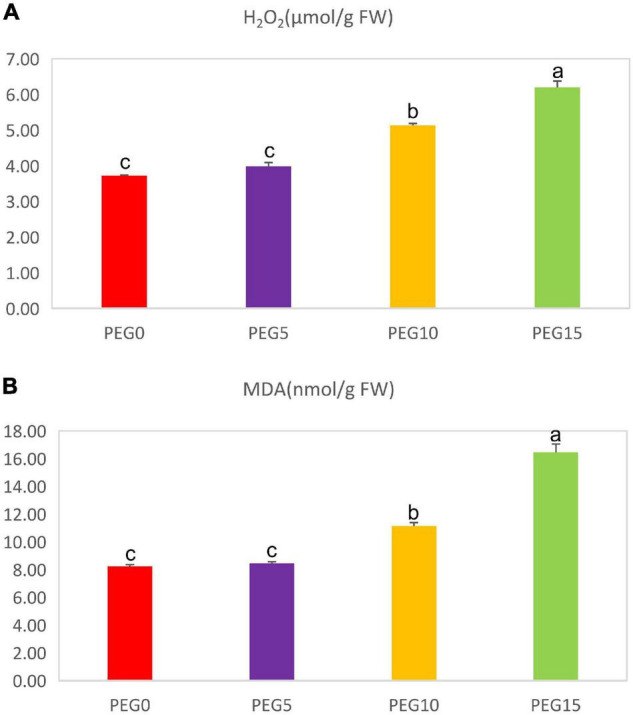
Changes of the malondialdehyde and hydrogen peroxide contents under different drought stress conditions. **(A)** The content of H_2_O_2_; **(B)** the content of malondialdehyde (MDA). Different letters indicate the significant difference at the 0.05 level.

The MDA and H_2_O_2_ contents were also determined in various drought treatments. The contents of MDA and H_2_O_2_ were dramatically increased with the increase of PEG6000 concentrations (Figure 5). Under 5% PEG6000 condition, they had no significant changes in comparison with the control. However, under 10 and 15% PEG6000 conditions, the MDA content was increased by 35.67 and 100.22%, and the H_2_O_2_ content was increased by 38.20 and 66.86%, respectively.

### RNA Sequencing, *de novo* Assembly, and Functional Annotation

To study the molecular mechanism of drought resistance in MCB, we selected PEG0 and PEG15 treatments to conduct the transcriptome analysis of six samples in this study. A total of 40.69 Gb clean data were obtained and the clean data of each sample were more than 6.07 GB and the percentage of Q30 base was more than 95.6% (Supplementary Table 2). All the clean data were *de novo* assembled by Trinity, and then the assembly results were optimized and filtered (TransRate^5^; CD-HIT^6^) and evaluated (BUSCO, Benchmarking Universal Single-Copy Orthologs^7^). We obtained 1,19,150 transcripts (total base, 12,38,02,946 bp; average length, 1039.05 bp; N50, 1,668 bp) and 71,785 unigenes (total base, 6,19,82,181 bp; average length, 863.44 bp; N50, 1,473 bp) (Supplementary Table 3). All the transcripts were aligned against NR, Swiss-Prot, Pfam, COG, GO, and KEGG databases (Supplementary Figure 1). Cellular process and metabolic process were most enrichment in “biological process” (BP) and membrane part and cell part were the most in “cellular component” (CC), and binding and catalytic activity was significant enrichment in “molecular function” (MF) (Supplementary Figure 2). A total of 11,394 unigenes aligned with the top20 classifications and the pathways were divided into six categories including environmental information processing, metabolism, cellular processes, genetic information processing, organismal systems, and human diseases (Supplementary Figure 3).

### Identification of Differentially Expressed Genes

**FIGURE 6:**
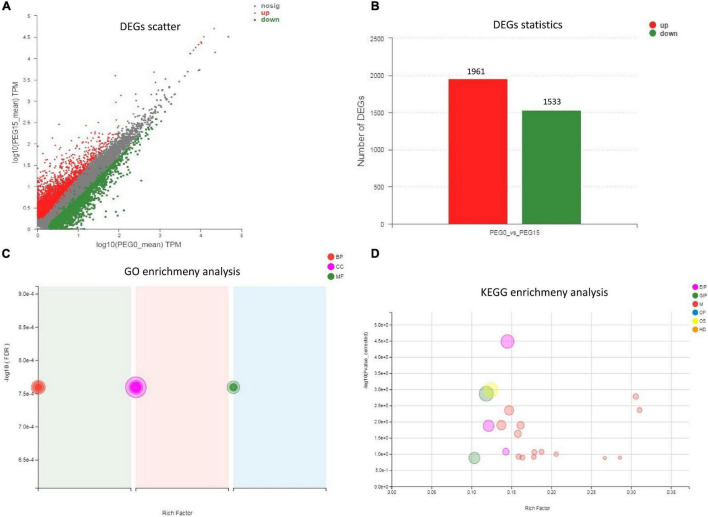
Differentially expressed genes (DEGs) statistics and functional enrichment analysis. **(A)** DEGs scatter analysis; **(B)** DEGs statistical analysis; **(C)** Gene Ontology (GO) enrichment analysis; **(D)** Kyoto Encyclopedia of Genes and Genomes (KEGG) enrichment analysis.

According to the expression of unigenes/transcripts among different samples, analysis of expression distribution, venn, correlation, and PCA were performed (Supplementary Figure 4). Further, to study the drought resistance mechanism of MCB, the DEGs analysis between PEG0 and PEG15 was conducted. DESeq2 (Michael et al., 2014) software was employed for DEGs analysis, and the screening threshold was: | log2FC| > = 1 and *p*-adjust < 0.05. A total of 3,494 DEGs were identified between the two treatments, including 1,961 up-regulated and 1,533 down-regulated unigenes (Figures 6A,B). GO enrichment showed the DEGs were involved in “lignin metabolic process,” “metal ion transport,” “pectin catabolic process,” “cell wall,” “oxidoreductase activity, acting on diphenols and related substances as donors, oxygen as acceptor,” “galactosyltransferase activity,” etc. (Figure 6C). KEGG enrichment revealed that the DEGs were associated with “plant hormone signal transduction,” “brassinosteroid biosynthesis,” “plant–pathogen interaction,” “starch and sucrose metabolism,” “phenylpropanoid biosynthesis,” and “pentose and glucuronate interconversions,” etc. (Figure 6D and Table 1).

**TABLE 1:** The metabolic pathways involved in response to drought stress in *Mesona Chinensis* Benth (MCB).

Pathway id	Description	Gene num	*P*-value corrected
map04075	Plant hormone signal transduction	63	3.23273E-05
map04626	Plant–pathogen interaction	70	0.001017808
map03010	Ribosome	75	0.001335686
map00905	Brassinosteroid biosynthesis	11	0.001618405
map00902	Monoterpenoid biosynthesis	9	0.004288898
map00940	Phenylpropanoid biosynthesis	31	0.004364363
map00500	Starch and sucrose metabolism	31	0.01249589
map00040	Pentose and glucuronate interconversions	20	0.012683802
map04016	MAPK signaling pathway – plant	44	0.013092807
map00052	Galactose metabolism	18	0.023076204

### Plant Hormone Signal Transduction in Response to Drought Stress

**FIGURE 7:**
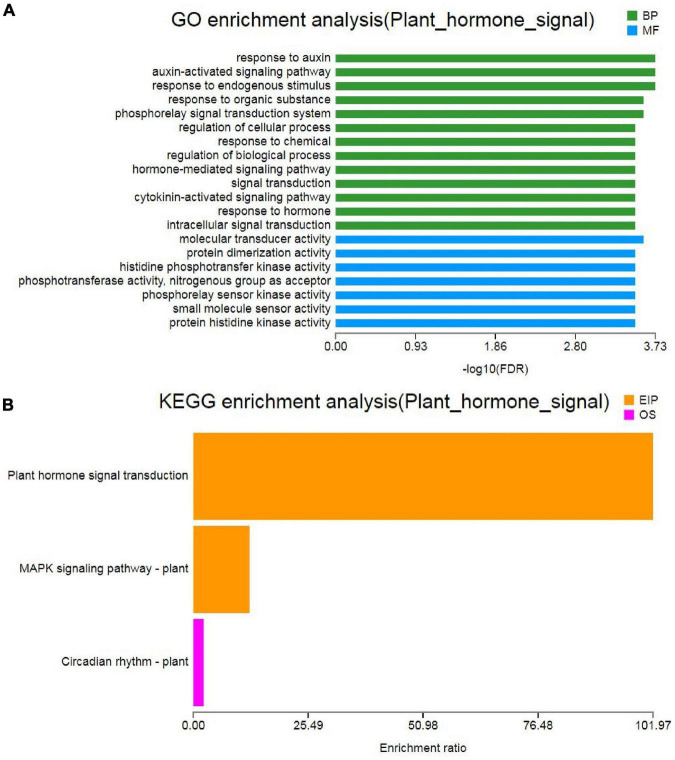
Gene Ontology and KEGG analysis of the “plant hormone signal transduction” pathway. **(A)** GO analysis of the “plant hormone signal transduction” pathway. **(B)** KEGG analysis of the “plant hormone signal transduction” pathway.

**FIGURE 8:**
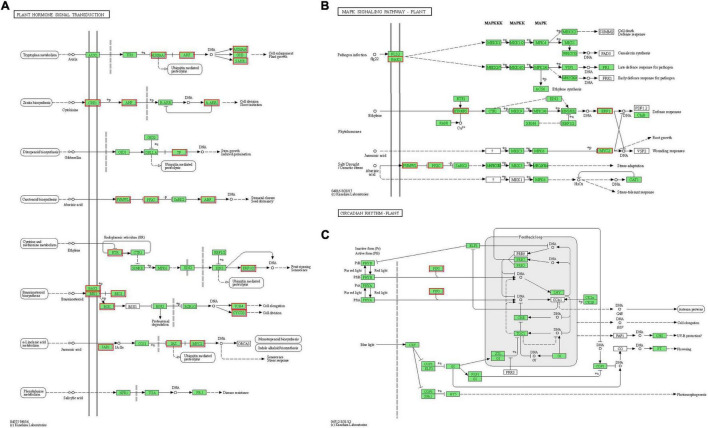
Differentially expressed genes involved in plant hormone signal transduction pathway. **(A)** The DEGs involved in the “Plant hormone signal transduction” pathway. **(B)** The DEGs involved in the “MAPK signaling pathway-plant” pathway. **(C)** The DEGs involved in the “Circadian rhythm-plant” pathway. The red boxes indicated the DEGs were involved in different hormone signal transduction pathways.

**FIGURE 9:**
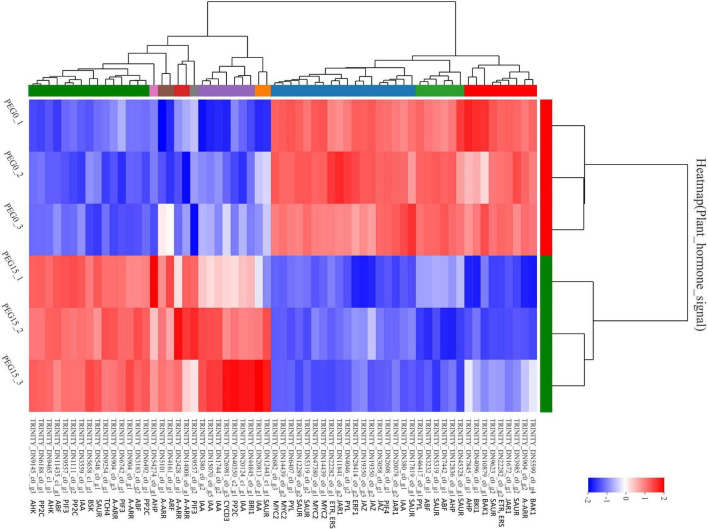
The heat map of the DEGs involved in the “plant hormone signal transduction” pathway.

Hormones play important roles in plant abiotic stress (Ryu and Cho, 2015). GO analysis of “plant hormone signal transduction” showed that the biological process and molecular function were the most enrichment categorizations (Figure 7A). Based on the KEGG analysis, we found that a total of 63 DEGs were enriched and showed close association with this pathway. Moreover, 16 DEGs were involved in “MAPK signaling pathway-plant” and 3 DEGs were involved in “circadian rhythm-plant” (Figure 7B). In the “plant hormone signal transduction” pathway, the 63 unigenes were mainly involved in the metabolism pathways of brassinosteroid (BR), ABA, ethylene (ETH), auxin (IAA), cytokinin (CTK), gibberellin (GA), and JA, including ethylene-responsive TF ERF, ABA receptor PYR/PYL, Auxin-responsive protein SAUR, Jasmonoyl-L-amino acid synthetase JAR4, etc. (Figure 8A and Supplementary Table 4). In the “MAPK signaling pathway-plant” pathway, the 16 DEGs were associated with BAK1, ETR/ERS, ERF, MYC2, PYR/PYL, and PP2C (Figure 8B and Supplementary Table 5). In the “circadian rhythm-plant,” 3 DEGs were related to PIF3 (Figure 8C and Supplementary Table 6). Therefore, it was inferred that various DEGs were involved in the “plant hormone and signal transduction” pathway in response to drought stress. In addition, among these DEGs, 33 unigenes were down-regulated and the remaining 30 unigenes were up-regulated under drought stress (Figure 9). These suggested that the expression of related genes in “plant hormone signal transduction,” “MAPK signaling pathway-plant,” and “circadian rhythm-plant” pathways might be closely related to drought response in MCB. In particular, ERF1 inhibited the production of MYC2 metabolites and MYC2 also inhibited the production of ERF1 metabolites in the “MAPK signaling pathway-plant” pathway (Figure 8B).

### Brassinosteroid Biosynthesis in Response to Drought Stress

**FIGURE 10:**
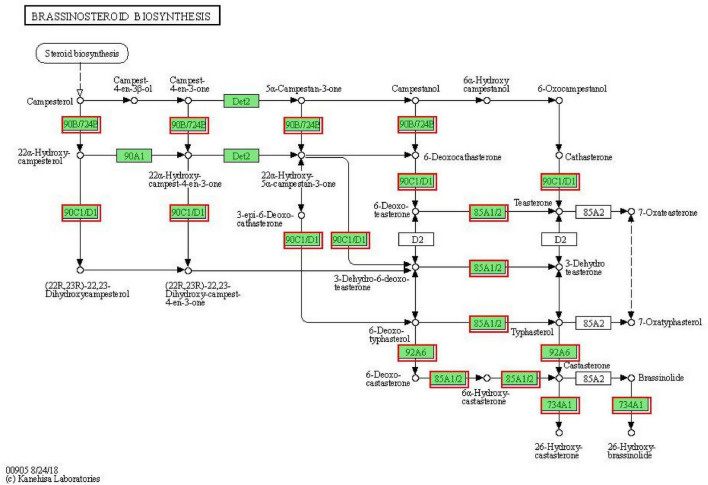
Differentially expressed genes involved the “brassinosteroid biosynthesis” pathway. The red boxes indicated the DEGs were involved in the “brassinosteroid biosynthesis” pathway.

**FIGURE 11:**
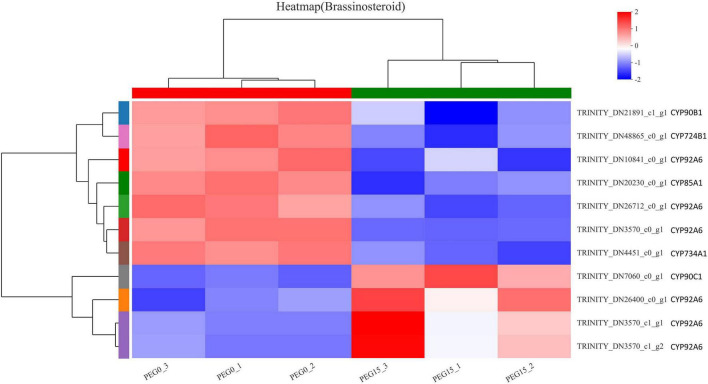
The heat map of the DEGs involved in the “brassinosteroid biosynthesis” pathway.

Brassinosteroids (BRs) regulate many physiological and developmental processes in plants, such as leaf expansion, cell elongation, flower development, light morphogenesis, stomatal development, and male sterility, and increase the plant tolerance to various kinds of abiotic stresses like drought, salinity, temperature, and heavy metal, etc. (Sharma et al., 2022). Combined with the results of the KEGG analysis of DEGs and “Plant hormone signal transduction,” we found that brassinosteroid biosynthesis might play important roles in response to drought stress in MCB. Therefore, to further reveal that the brassinosteroid biosynthesis might be involved in drought stress response, the genes related to brassinosteroid biosynthesis were analyzed. As shown in Figure 10, a total of 11 DEGs were found in the “brassinosteroid biosynthesis” pathway and involved in multiple steps of brassinosteroid biosynthesis. The 11 DEGs were *CYP90B1, CYP724B1, CYP92A6, CYP85A1, CYP734A1*, and *CYP90C1*. Of these, 7 unigenes were down-regulation, while 4 unigenes were up-regulation under drought stress conditions (Figure 11). It was indicated that these genes might be involved in drought stress.

### Differentially Expressed Transcription Factors Involved in Response to Drought Stress

**FIGURE 12:**
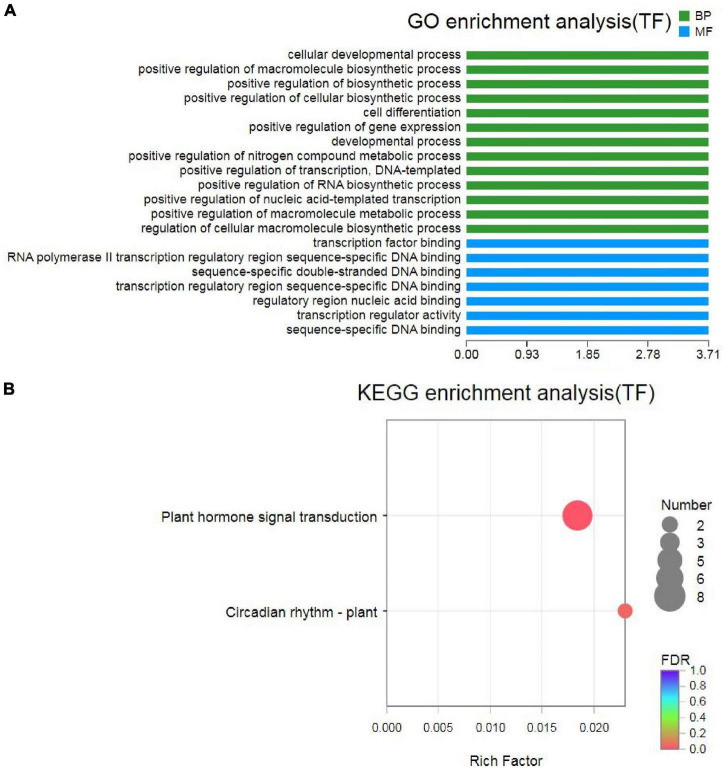
Gene Ontology and KEGG analysis of TFs. **(A)** GO analysis of TFs; **(B)** KEGG analysis of TFs.

Transcription factors also play important roles in response to drought stress (Mun et al., 2017). In this study, a total of 1,294 and 2,667 TFs were identified in the unigenes and transcripts of MCB, which were distributed in various TF families, such as *bHLH*, *C2C2*, *AP2/ERF*, *NAC*, *bZIP*, *WRKY*, *MYB_superfamily*, etc. (Supplementary Table 7). In total, 204 TFs were identified in DEGs and distributed in 24 families, including *bHLH*, *MYB_superfamily*, *C2C2*, *AP2/ERF*, *NAC*, *bZIP*, *WRKY*, and so on (Supplementary Table 8). GO enrichment analysis revealed that these 204 TFs were involved in “cellular developmental process,” “positive regulation of macromolecule biosynthetic process,” “cell differentiation,” etc. in BP and “TF binding,” “transcription regulatory region sequence-specific DNA binding,” “transcription regulator activity,” etc. in MF (Figure 12A). Further KEGG analysis unveiled that 8 TFs (*TRINITY_DN3232_c0_g1, TRINITY_DN4161_ c0_g1, TRINITY_DN3183_c0_g2, TRINITY_DN28414_ c0_g2, TRINITY_DN11435_c1_g1, TRINITY_DN2608_c0_g1, TRINITY_DN6742_c0_g1*, and *TRINITY_DN9557_c0_g1*) and two TFs (*TRINITY_DN6742_c0_g1* and *TRINITY_ DN9557_c0_g1*) were associated with “plant hormone signal transduction” and “circadian rhythm-plant” pathways, respectively (Figure 12B and Supplementary Table 9). It was indicated that these TFs might be closely related to drought stress.

### qPCR Verification

To verify the accuracy of transcriptome data, we selected 12 genes (eight protein-coding genes and four TFs) for qPCR analysis. The results showed that the fold changes of nine genes were in accordance with those of the transcriptome data. However, the fold changes of *CYP90B1*, *CYP85A1*, and *CYP734A1* differed (Table 2).

**TABLE 2:** qPCR verification.

Gene Id	KO name	RNA-seq (fold change)	qPCR (fold change)
*TRINITY_DN26712_c0_g1*	CYP92A6	0.07	0.46
*TRINITY_DN7060_c0_g1*	CYP90C1, ROT3	2.09	9.52
*TRINITY_DN21891_c1_g1*	CYP90B1, DWF4	0.49	1.13
*TRINITY_DN48865_c0_g1*	CYP724B1, D11	0.28	0.58
*TRINITY_DN3570_c1_g1*	CYP92A6	150.85	13.88
*TRINITY_DN26400_c0_g1*	CYP92A6	2.73	8.96
*TRINITY_DN20230_c0_g1*	CYP85A1, BR6OX1	0.47	1.40
*TRINITY_DN4451_c0_g1*	CYP734A1, BAS1	0.22	1.62
*TRINITY_DN3232_c0_g1*	ABF	0.39	0.50
*TRINITY_DN4161_c0_g1*	K14486, ARF	2.43	2.76
*TRINITY_DN3183_c0_g2*	ABF	2.14	3.53
*TRINITY_DN28414_c0_g2*	ERF1	0.20	0.47

## Discussion

### Drought Stress Significantly Limited the Growth of *Mesona chinensis* Benth

Drought is a global problem that restricts the construction of the ecological environment and the distribution and productivity of plants. The effects of drought stress on plants are finally reflected in plant growth and development. Drought stress significantly affects the accumulation and distribution of plant biomass (Kashif, 2011). The drought adaptability of plants is closely related to the biomass distribution between shoot and root (Xu et al., 2015). Water stress significantly decreases the crop growth rate and fresh weight in tobacco (Shtereva et al., 2017). Similarly, in wheat, the plant root and shoot dry weight, plant height, leaf area, and yield are affected by drought (Hammad and Ali, 2014). In this study, drought stress significantly affected the fresh weight of the single plant, root, and above-ground parts of MCB (Figures 1, 2). Our results were consistent with the previous report (Yan et al., 2011).

The chlorophyll content is the most abundant pigment in the biosphere, which is directly related to plant photosynthesis, growth, and development and is an important indicator for the degree of stress (Kamble et al., 2015). Chlorophyll a (Chla) and chlorophyll b (Chlb) are the main components of chlorophyll. In the present study, the contents of Chla, Chlb, and Chla + b were increased with the aggravation of drought stress (Figures 3A–C). It was indicated that the increase of chlorophyll content might help to protect the photosynthetic system of MCB to reduce the damage of drought stress.

Soluble sugar (SS) is an osmoregulation substance actively accumulated by plants under stress, which can not only reduce the osmotic potential of leaf cells but also improve the water absorption capacity of plants, so as to reduce drought stress damage (Yancey, 2001). Drought-induced or drought-inhibited soluble protein (SP) is usually employed to evaluate the drought tolerance of plants (Kang et al., 2005). Studies have demonstrated that the accumulation of SP and SS is one of the most important responses of plants to drought stress (Silva et al., 2010; Li et al., 2014). In our investigation, the SS and SP contents were dramatically accumulated with the increasing PEG concentrations (Figures 3D,E). It was in line with the previous findings (Hu et al., 2015). In addition, the soluble pectin content was also increased and had a positive correlation with drought stress (Figure 3F). Soluble pectin is an important index to measure the quality of MCB, therefore it might also be used as a drought resistance indicator of MCB.

### Drought Stress Induced Anti-oxidative Defense and Osmotic Adjustments

Drought stress interrupts the balance of the ROS (e.g., superoxide and H_2_O_2_) production and antioxidant defense mechanism in plants (He et al., 2017), and an excessive formation of ROS can result in oxidative damage in plants (Sharma et al., 2012). Multiple antioxidant enzymes, e.g., SOD, POD, and CAT, are associated with the response of a plant to drought stress, and the production of the antioxidant enzymes is considered as a protective mechanism of plants against drought stress (Li and Yi, 2012). SOD is the key enzyme in the active oxygen scavenger system because it catalyzes the dismutation of superoxide free radicals into H_2_O_2_ and O_2_, and POD and CAT further convert H_2_O_2_ into H_2_O and O_2_, and then the damage caused by ROS is eliminated from plants (Wu et al., 2012; Hu et al., 2015). Total antioxidant capacity (TAC) reflects an integration activity of several antioxidants and is an important parameter in the analysis of biological matrices (Jaberiea et al., 2020). Here, the activities of SOD, CAT, and TAC were significantly increased with the aggravation of drought stress, while the POD activity was increased in PEG5 and PEG10, then decreased in PEG15 (Figure 4). It was inferred that POD, SOD, and CAT could effectively scavenge free radicals to reduce drought damage to MCB plants.

Malondialdehyde (MDA), a product of lipid peroxidation and H_2_O_2_, is generally used as a stress biomarker for the detection of membrane lipid peroxidation in plants (Yonny et al., 2017). The content of MDA is positively correlated with the damage degree of the plant plasma membrane (Zhong et al., 2021). Previous studies have reported an increased amount of MDA and H_2_O_2_ in response to drought stress (Cao et al., 2017). Drought stress induces a larger production of MDA in the leaves of tobacco (Khan et al., 2019). In our study, the MDA and H_2_O_2_ contents were increased with the increase of PEG6000 concentrations (Figure 5). These findings were supported by Yang et al. (2015), indicating that the plant plasma membrane was damaged to a certain extent and the lipid peroxidation of the plasma membrane was gradually enhanced.

### Plant Hormone Signal Transduction Involved in Response to Drought Stress

Phytohormone may be the crucial regulator in response to the harmful effects of various stress types and the coordination between the different hormone signaling is flexible in stress types and intensity and plant types under stress (Suzuki, 2016). ABA is an important plant hormone known to mediate the responses to drought stress, which results in the expression of genes related to the specific condition by initiating the cell signaling pathways (Lee and Luan, 2012). In the ABA signaling pathway, PYR/PYL/RCAR is the receptor, PP2C acts as a negative regulator, while SnRK2 acts as a positive regulator (Zhang et al., 2006; Khan et al., 2019). Under normal growth conditions, the contents of ABA and PYR/PYL/RCAR protein in plants are at a low level, and PP2C phosphatase binds to SnRK2 kinase and inhibits its activity, and the ABA signal remains silent; Whereas, the pathway is triggered by the binding of ABA to the PYR/PYL/RCAR receptor, which then sequesters the PP2C, allowing the downstream SnRK2s to activate or inactivate the targets by phosphorylation, which include KAT1, SLAC1, the NADPH oxidase AtrbohF, and several *b-ZIPs* (Joshi-Saha et al., 2011). In this study, drought stress induced the differential expression of *PYL*, *PP2C*, and *ABF.* In this pathway, 3 PYL genes were down-regulation, while 4 PP2C genes were up-regulation, and 4 ABF genes (2 down-regulation and 2 up-regulation) were identified. These suggested that the down-regulation of *PYL* might enhance the up-regulation of *PP2C*, activate *SnRK2s*, promote the expression of *ABF*, and finally produce a series of physiological and biochemical anti-stress reactions.

Jasmonic acid and its derivatives activate the signaling pathways similar to ABA (Riemann et al., 2015). Studies have suggested that jasmonate ZIM (zinc-finger inflorescence meristem) domain (JAZ) proteins in the JA signaling pathway may be involved in the generation of a response to drought (Liu et al., 2017). JAZ family members act as repressors of JA signals, which directly interact with the basic helix-loop-helix leucine zipper-type (*bHLH zip-type*) factor *MYC2* (Melotto et al., 2008). *MYC2*, a key transcriptional activator that regulates JA-dependent transcriptional reprogramming (Boter et al., 2004). In this pathway, the transcription repressor JAZ protein interacts with the downstream positive regulatory factor *MYC2*, so as to inhibit the regulation of MYC2 on downstream gene expression (Hou et al., 2010). When plants are subjected to environmental stresses, the stress signals improve the production of JA in peroxidase, and then the JA is transported to the cytoplasm to form jasmonoyl-isoleucine (JA-Ile) by JA-Ile synthase (JAR1); JA-Ile promotes the ubiquitin ligase complex (SCF^coi1^) to form a complex with JAZ, and then 26S protease hydrolyzes the JAZ protein, releasing MYC2, which can be combined with the G-box of downstream target genes, thus affecting the expression of downstream genes (Wasternack and Strnad, 2016; Wasternack and Song, 2017). Here, under drought stress conditions, 3 *JAR1* genes and 3 *JAZ* genes were down-regulated while 4 *MYC2* genes were up-regulated, indicating that the down-regulation of *JAR1* and *JAZ* genes might promote the dissociation and release of MYC2 and then lead to the stress response of MCB.

Auxin is an important regulatory signal in the process of plant growth. It mainly plays a role in the vigorous tissue, but it is also considered to be a negative regulator of plant drought resistance (De and Jürgens, 2007). In the auxin signal transduction pathway, the extracellular receptor senses the changes of the external environment, the signal is transmitted to the cell through a variety of related pathways, then the nuclear auxin receptor receives the signal, and the complex process of gene expression regulation starts (Chen et al., 2007). Plant auxin primary response genes include three main categories: *AUX/IAA*, *SAUR*, and *GH3* gene families (Liscum and Reed, 2002). *Small auxin up-RNA* (*SAUR*) genes promote cell expansion (Chae et al., 2012). SAUR proteins provide a mechanistic link between auxin and plasma membrane H^+^-ATPases in *Arabidopsis thaliana* and interact with the PP2C-D subfamily of type 2C protein phosphatases (Spartz et al., 2014). The plant *GH3* gene family is a typical auxin-responsive gene family, which is involved in the auxin signaling pathway, light signaling, and plant defense responses (Li et al., 2008). The promoter of plant *GH3* usually contains auxin response element (*AuxRE*) TGTCTC sequence, which can specifically bind to auxin response factor (*ARF*) (Ulmasov et al., 1997). Auxin signal transduction is mainly completed through the interaction between *ARF* and auxin/indole-3-acetic acid (*AUX/IAA*) (Piya et al., 2014). When the auxin concentration in plants is at a low level, *AUX/IAA* binds to *ARF* to prevent *ARF* from activating auxin-related gene transcription; When auxin concentration increases, *AUX/IAA* binds to auxin receptor *TIR* and is degraded, releasing *ARF* to regulate the expression of auxin response-related genes (Guilfoyle and Hagen, 2007). In the present study, the *AUX/IAA*, *ARF*, and *SAUR* genes responded to drought stress in this pathway. Among them, 6 up-regulation and 1 down-regulation genes were involved in AUX/IAA, 1 up-regulation gene was in involved ARF, and 2 up-regulation and 7 down-regulation genes were involved in SAUR. It was inferred that the expression of *AUX/IAA* contributed to the expression and release of ARF protein and the expression of SAUR, thus affecting plant growth of MCB.

Phytohormone ethylene affects a series of developmental processes and stress-resistance responses in plants. In *A. thaliana*, the ethylene signal transduction pathway has been well studied. Briefly, under the action of Cu^+^, ethylene molecules bind to ethylene receptors (ETR1, ERS1, ETR2, ERS2, and EIN4), resulting in the inactivation of the receptor CTR1 complex. The inactivated CTR1 no longer phosphorylates the downstream EIN2, consequently, the EIN2 is activated because it is not degraded. Then, the carboxyl end of EIN2 protein is cleaved and dissociated and enters the nucleus (Ji and Guo, 2013). EIN2 center may promote the accumulation of EIN3/EIL1 in the nucleus by inhibiting the ubiquitination and degradation of EIN3/EIL1 mediated by EBF1/2 protein (Guo and Ecker, 2003). EIN3/EIL1 activates downstream target gene expression such as *ethylene response factor 1* (*ERF1*) at the transcriptional level. Meanwhile, *ERF1*, as a TF also activates the expression of downstream target genes. Subsequently, a large number of downstream ethylene response genes are activated at the transcriptional level, resulting in ethylene reaction (Zhao and Guo, 2011). In our investigation, 2 *ETR* genes and 1 *ERF1* gene were induced to be differentially expressed by drought stress and they were down-regulated compared to the normal conditions. *ERFs* are unique to plants and belong to the AP2/EREBP-type TFs, which play a role of *trans*-acting factors at the last step of ethylene signaling (Klee and Giovannoni, 2011; Xiao et al., 2013). *ETR* is one of the key elements in the ethylene signal transduction pathway, which plays an important role in regulating plant growth and development and resisting stress (Bisson and Groth, 2010). Therefore, it was indicated that *ETR* and *ERF1* might be involved in responses to drought stress of MCB.

Cytokinins play important roles in plant growth and development and stress tolerance (Sakakibara, 2006). Cytokinin signal transduction involves a multistep two-component system consisting of *Arabidopsis* histidine phosphotransfer proteins (AHP), histidine kinases (AHK), response regulators (ARRs), and so on (Hwang and Sheen, 2001). Cytokinin receptors AHK2, AHK3, and CRE1 bind to cytokinin, self phosphorylate, and transfer the phosphate groups from the histidine residues conserved in kinase region to aspartate residues conserved in signal receiving region; The phosphate groups on aspartic acid are transferred to *Arabidopsis* histidine-phosphotransfer proteins (AHPs) in the cytoplasm; The phosphorylated AHPs enter the nucleus and transfer the phosphate groups to *Arabidopsis* response regulators (ARRs), regulating the downstream cytokinin reaction, so as to produce a series of biochemical effects and regulate plant growth and development (Sakakibara, 2006; Muller and Sheen, 2007). In this pathway, our results exhibited 3 DEGs (1 up-regulated and 2 down-regulated), 6 DEGs (5 up-regulated and 1 down-regulated), and 2 DEGs (up-regulated) were in AHP, A-ARR, and AHK, respectively. *OsAHPs* function as positive regulators of the cytokinin signaling pathway and the expression of *OsAHP1* and *OsAHP2* are inhibited by drought stress (Sun et al., 2014). The mutations in *a-arr5*, *a-arr6*, or *a-arr7* lead to enhanced cold tolerance, suggesting that the *A-ARR5*, *A-ARR6*, and *A-ARR7* negatively regulate cold stress responses (Jeon et al., 2010). *AHK1* is considered to be a positive regulator of osmotic stress signaling because the overexpression of *AHK1* shows enhanced stress tolerance, while *ahk1* mutant is sensitive to osmotic stress (Tran et al., 2007; Wohlbach et al., 2008). Thus, it was indicated that the 2 down-regulated *AHP* genes, 1 down-regulated *A-ARR* gene, and 2 down-regulated *AHK* genes might be involved in drought resistance response in MCB.

Brassinolide (BR) is an important steroidal hormone and plays an important role in promoting plant growth and improving plant stress resistance (Hao et al., 2017). Within the BR signal transduction, BR is recognized by membrane receptor BRI1 (Wang et al., 2012), which dissociates its inhibitor, BRI1 associated receptor kinase 1 (BAK1), from the plasma membrane; At the same time, the BRI1-associated receptor kinase 1 (BAK1) on the membrane forms an allotropic polymer with BRI1; The BRI1-BAK1 complex inhibits the activity of downstream brassinosteroid insensitive 2 (BIN2) kinase and activates the activity of BRI1 suppressor 1 (BSU1) phosphatase, which changes the phosphorylation level of TFs BES1 and BZR1; BES1 and BZR1 directly bind to the promoter of BRs related genes and regulate their expression positively or negatively (Belkhadir et al., 2006). BAK1 (BRI1 associated receptor kinase 1) is a receptor kinase rich in Leu repeats, which can regulate brassinolide receptor BRI1 and make plants resistant to bacterial antigens (Chinchilla et al., 2007). The *bak1-bkk1* mutants show plant lethal phenotype, callose deposition, ROS accumulation, and even spontaneous cell death under poor growth conditions, indicating that BAK1 and BKK1 positively regulate the plant growth pathway dependent on BR and negatively regulate the apoptosis pathway independent of BR (He et al., 2007). Brassinosteroid signaling kinase (BSKs) family members can interact with and be phosphorylated by BRI1 (Tang et al., 2008). CYCD is a crucial regulatory factor in the G1 phase and can promote the transformation of cells from the G1 phase to the S phase, which plays an important role in regulating cell division, size, quantity, and organ morphogenesis (Bisbis et al., 2006). *TCH4* gene, encoding xyloglucan endoglycosylase, can transfer xyloglucan in the cell wall during plant morphogenesis and affect the formation and degradation of the cell wall, thus playing an important role in plant secondary growth, disease resistance, and stress resistance (Xu et al., 1995; Zhang et al., 2008). In our study, the results showed that the *BRI* (included 1 DEGs, up-regulation), *BKI* (included 1 DEGs, down-regulation), *BAK* (included 2 DEGs, down-regulation), *BSK* (included 1 DEGs, up-regulation), *TCH4* (included 1 DEGs, up-regulation), and *CYCD3* (included 1 DEGs, up-regulation) were found to be differentially expressed under drought stress conditions. In this pathway, the up-regulated expression of *BRI* might promote the *BSK* expression, leading to the up-regulated expression of *TCH4* and *CYCD3*, finally resulting in the drought stress responses of MCB. Overall, the genes involved in different steps of the “plant hormone signal transduction” pathway were significantly differential expression under drought stress, indicating that when MCB encountered drought stress, the MCB plants might adapt to drought conditions by regulating the expression of related genes in the “plant hormone signal transduction” pathway.

In addition, in the EHT and JA signaling pathways, ethylene-responsive element-binding factors (*ERFs*) form a plant-specific transcriptional factor superfamily of 147 members in *A. thaliana* (Nakano et al., 2006) and influence a number of developmental processes and are also important for adaptation to biotic or abiotic stresses (Cheng et al., 2013). The expression of multiple members of the APETALA2/ETHYLENE RESPONSE FACTOR (AP2/ERF) family requires the synergistic effect of ETH and JA signals (Zhou et al., 2010). *MYC2*, as the core TF of the JA signal transduction pathway, is involved in regulating plant growth, development, and stress response. In this study, ERF1 inhibited the production of MYC2 metabolites and MYC2 also inhibited the production of ERF1 metabolites in the “MAPK signaling pathway-plant” pathway (Figure 8B). It was demonstrated that ERF1 was proposed to be an integrator of ETH and JA defense responses (Lorenzo et al., 2003). This suggested that the phytohormones (ETH and JA) might coordinate responses to drought stress in MCB.

### Brassinosteroid Biosynthesis Involved in Response to Drought Stress

As mentioned above, the KEEG results of DEGs revealed that brassinosteroid biosynthesis was also one of the most significant enrichment pathways (Table 1). Therefore, we speculated that the brassinosteroid biosynthesis might play important role in response to drought stress in MCB. Our data showed that 11 unigenes, including *CYP90B1, CYP90C1, CYP85A1, CYP724B1, CYP734A1*, and *CYP92A6* were involved in brassinosteroid biosynthesis. In *A. thaliana*, the transcript levels of *CYP90* and *CYP85* genes are down-regulated by brassinolide, which is the end product of the brassinosteroid biosynthesis pathway. *CYP90C1* and *CYP85A1* mRNAs are more abundant in roots, whereas *CYP90B1* is ubiquitously expressed (Bancos et al., 2002). *CYP85A1* and *CYP85A2* are two *CYP85A* gene members in *A. thaliana* and *CYP85A2* mediates the conversion of castasterone to brassinolide and *CYP85A1* is essential for the initiation of female gametogenesis in *A. thaliana* (Pérez-España et al., 2011). *CYP724B1* gene plays a role in BR synthesis and may be involved in the supply of 6-deoxotyphasterol and typhasterol in the BR biosynthesis network and is associated with the internode elongation and seed development in rice (Tanabe et al., 2005). *CYP734As* as a key gene in inactivated brassinosteroids, regulate plant growth and development (Nakamura et al., 2005). *CYP734A7* encodes a C-26 hydroxylase of brassinosteroids and is probably involved in brassinosteroid catabolism (Ohnishi et al., 2006). ZmCYP92A1 protein has a close relationship with flavonoids 3-monooxygenase of the CYP75 subfamily and is involved in abiotic stresses defense (Duan and Song, 2018). In the present study, the 11 unigenes were differentially expressed under drought stress. It was inferred that the expression of these genes might be closely related to the brassinosteroid biosynthesis. The differential expression of these genes might lead to the change of brassinolide content, thus affecting the growth and development of MCB.

### Transcription Factors Involved in Response to Drought Stress

In this study, we found that 8 DEGs (TFs) might be involved in response to drought stress in MCB (Supplementary Table 9). These TFs included *ABA-responsive element* (*ABRE*)*-binding transcription factor1* (*AREB1*) (*TRINITY_DN3232_c0_g1*), *auxin response factor* (*ARF9*) (*TRINITY_DN4161_c0_g1*), *abscisic acid-insensitive 5-like protein* (*ABI5*) (*TRINITY_DN3183_c0_g2*), *ethylene-responsive transcription factor ERF1b* (*ERF1b*) (*TRINITY_DN28414_c0_g2*), *phytochrome-interacting factors* (*PIF3*) (*TRINITY_DN9557_c0_g1*), etc. *AREB1* regulates ABRE-dependent ABA signaling that enhances drought tolerance in vegetative tissues (Fujita et al., 2005). *AREB1* is a basic domain/leucine zipper (bZIP) TF that binds to the ABA-responsive element (ABRE) motif in the promoter region of ABA-inducible genes; The bZIP proteins function to activate plant genes responsive to various physiological, developmental, and environmental signals, and the molecular mechanisms that may control gene-specific transcriptional activation involve post-translational modification, heterodimerization, and nuclear localization (Foster et al., 1994). *ABI5*, also belonging to the 13-member group A of *Arabidopsis* bZIP TFs (Jakoby et al., 2002) contributes to the induction of desiccation tolerance (Maia et al., 2014). Therefore, in this study, the *AREB1* and *ABI5* might activate ABA-inducible genes through post-translational modification, heterodimerization, or nuclear localization so as to respond to drought stress.

Auxin response factor*s* are involved in plant growth and developmental processes. *ARFs* are TFs that bind with specificity to TGTCTC auxin response elements (*AuxRE*) in promoters of the auxin response genes (Guilfoyle and Hagen, 2001). *ARFs* function in combination with Aux/IAA (auxin/indole acetic acid) repressors, which dimerize with ARF activators in an auxin-regulated manner (Guilfoyle and Hagen, 2007). ARF7 and ARF19 activators regulate the expression of the *ARF19* gene and a subset of auxin response genes (Wilmoth et al., 2005). *arf*7 *arf*19 double mutants have suggested that ARF7 plays a major role and ARF19 a lesser role in regulating a variety of auxin response genes in young seedlings (Okushima et al., 2005). In floral tissues, *arf6 arf8* double mutants show the reduced expression of *SAUR62-SAUR67* and *AUX/IAA* genes, indicating that ARF6 and ARF8 may activate at least some auxin response genes (Nagpal et al., 2005). The auxin-inducible *GH3* genes (*GH3.5* and *GH3.6*), which are thought to be activated by ARF8 and repressed by ARF17, are the candidate targets for ARF8 and ARF17 (Tian et al., 2004; Mallory et al., 2005). Here, the *ARF9* was found to be up-regulated under drought stress conditions. Previous evidence has demonstrated that the *ARF* transcript abundance is regulated by microRNAs at the posttranscriptional level (Rhoades et al., 2002; Zouine et al., 2014; Shen et al., 2015). Transcriptional regulation and post-transcriptional regulation may play an important role in controlling the levels of different ARF proteins in various cells and tissues and the target genes, containing the AuxRE sequences GAGACA or TGTCTC in their promoter regions may be the potential binding sites for the ARF proteins (Guilfoyle and Hagen, 2007).

In *A. thaliana*, the perception of light signals by the phytochrome (phy) family of sensory photoreceptors (phyA-phyE) begins an intracellular transduction process that leads to the changes in the expression of nuclear genes that affect plant growth and developmental responses (Rockwell et al., 2006). *PIF3*, a direct phytochrome reaction partner in the photoreceptor’s signaling network, is a G-box binding bHLH protein that interacts preferentially with the active form of phytochrome and is involved in controlling the expression of light-regulated genes such as *CCA1* and *LHY* (Martinez-Garcia et al., 2000). *PIF3* is firstly identified in a yeast two-hybrid screen for phyB-interacting proteins and subsequently found to bind conformer-specifically to the Pfr form of both phyA and phyB in a photoreversible fashion (Ni et al., 1999). In the present study, the MCB plantlets were exposed to the red light condition and the *PIF3* (*TRINITY_DN9557_c0_g1*) was up-regulated under drought stress conditions. These indicated that the *PIF3* not only responded to the red light but also might be involved in response to drought stress.

*ERF1b* acts as a transcriptional activator and binds to the GCC-box pathogenesis-related promoter element, which is involved in the regulation of gene expression during the plant development, and/or mediated by stress factors and by components of stress signal transduction pathways (Onate-Sanchez and Singh, 2002). Here, the *ERF1b* interacted with *MYC2* in the “MAPK signaling pathway-plant” pathway, indicating that *ERF1b* might be a key integrator of ETH and JA signals in the regulation of ETH/JA-dependent defenses (Lorenzo et al., 2003).

## Conclusion

**FIGURE 13:**
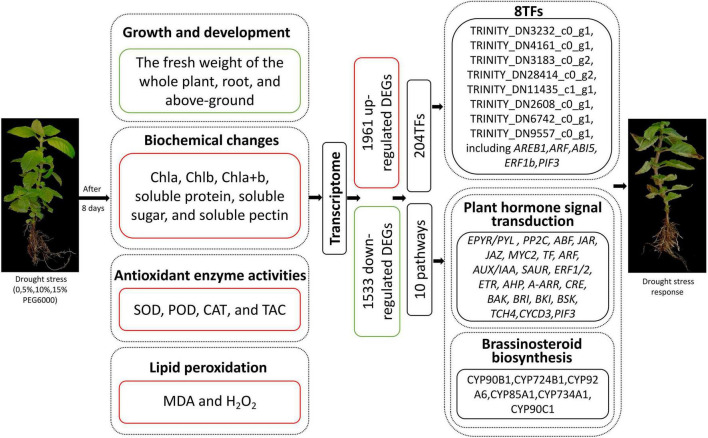
Overview of drought stress affecting the growth and development of MCB. Red boxes and words indicated up-regulation; green boxes indicated down-regulation.

Drought stress significantly limited the growth of MCB. The fresh weight of the whole plant, root, and above-ground was significantly decreased, while the contents of Chla, Chlb, Chla + b, soluble protein, soluble sugar, soluble pectin, SOD, CAT, TAC, MDA, and H_2_O_2_ were dramatically increased with the increasing concentrations of PEG6000. The POD activity increased then decreased with the increase of PEG6000 concentration. Transcriptomic analyses revealed a total of 1,961 up-regulated and 1,533 down-regulated unigenes were identified. KEGG enrichment demonstrated that these DEGs were associated with “plant hormone signal transduction,” “brassinosteroid biosynthesis,” “plant–pathogen interaction,” “starch and sucrose metabolism,” “phenylpropanoid biosynthesis,” and “pentose and glucuronate interconversions,” etc. In addition, a total of 204 TFs were identified in DEGs and 8 TFs were significantly enriched in the “plant hormone signal transduction” pathway. These suggested that the genes and TFs might play important roles in response to drought stress in MCB (Figure 13).

## Data Availability Statement

The original contributions presented in the study are publicly available. This data can be found here: National Center for Biotechnology Information (NCBI) BioProject database under accession number PRJNA777790.

## Author Contributions

DT: conceptualization, methodology, investigation, formal analysis, writing-original draft, and writing-reviewing and editing. DT, SQ, FW, and JM: funding acquisition. YiL, KW, YaL, and CQ: methodology and investigation. All authors approved the submitted version.

## Conflict of Interest

The authors declare that the research was conducted in the absence of any commercial or financial relationships that could be construed as a potential conflict of interest.

## Publisher’s Note

All claims expressed in this article are solely those of the authors and do not necessarily represent those of their affiliated organizations, or those of the publisher, the editors and the reviewers. Any product that may be evaluated in this article, or claim that may be made by its manufacturer, is not guaranteed or endorsed by the publisher.

## Footnotes

1
https://github.com/najoshi/sickle

2
https://github.com/jstjohn/SeqPrep

3
http://trinityrnaseq.sourceforge.net/

4
http://www.blast2go.com/b2ghome

5
http://hibberdlab.com/transrate/

6
http://weizhongli-lab.org/cd-hit/

7
http://busco.ezlab.org
