# Supplementary material for: Physio-Morphological, Biochemical and Transcriptomic Analyses Provide Insights Into Drought Stress Responses in Mesona chinensis Benth
Source: Front Plant Sci. 2022 Feb 10;13:809723. doi: 10.3389/fpls.2022.809723 (PMC8866654; doi:10.3389/fpls.2022.809723)
Supplement: Supplementary file 1 [file Data_Sheet_1.docx]

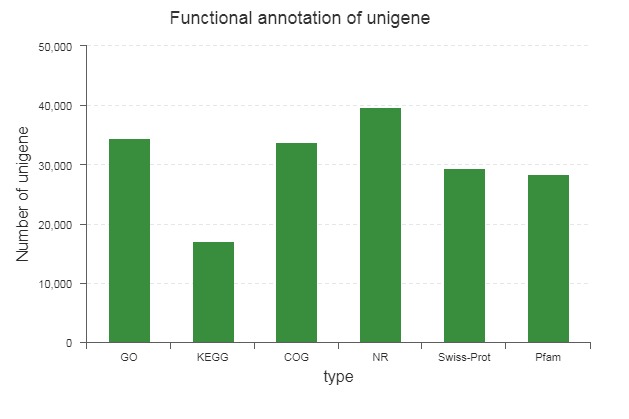
FIGURE S1 Functional annotation of unigenes.


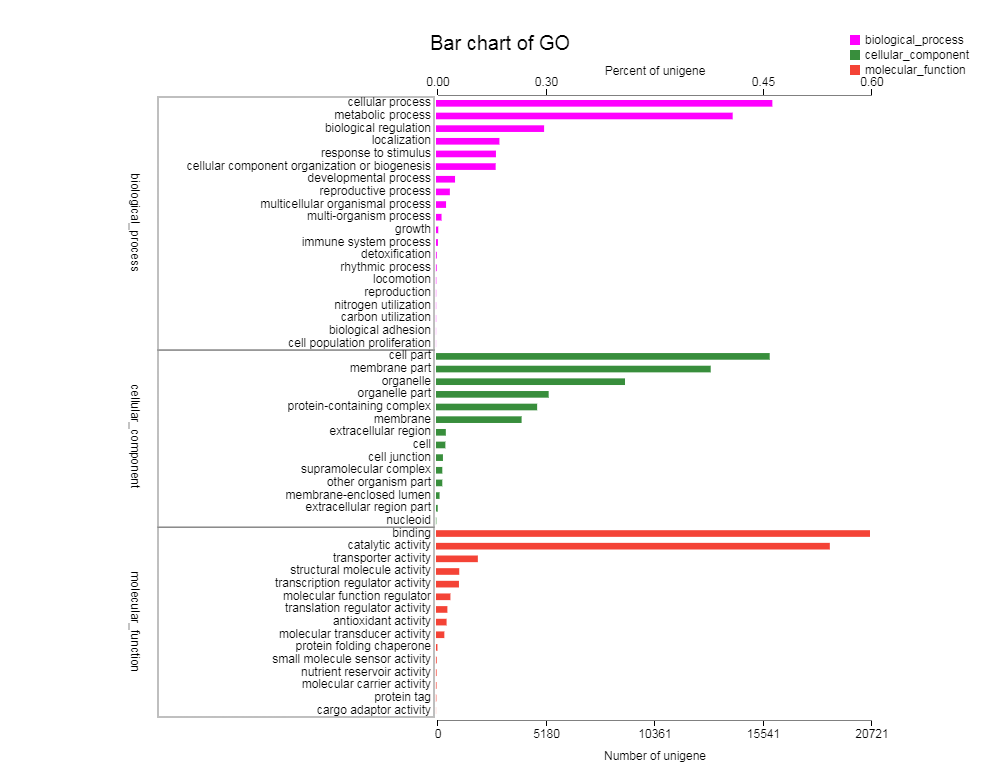


FIGURE S2 Analysis of the top20 GO terms.


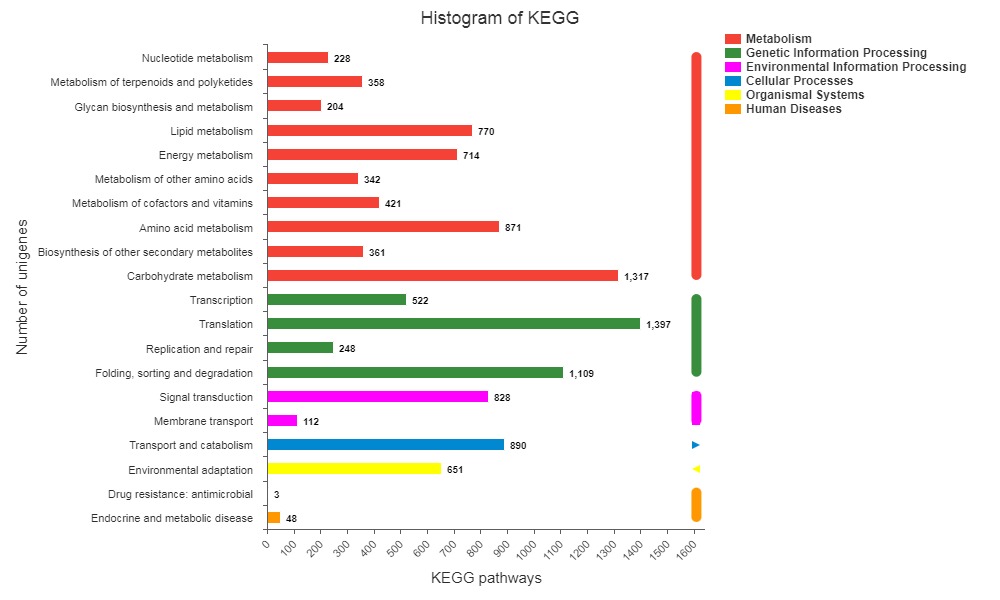


FIGURE S3 Analysis of the top20 KEGG pathways.


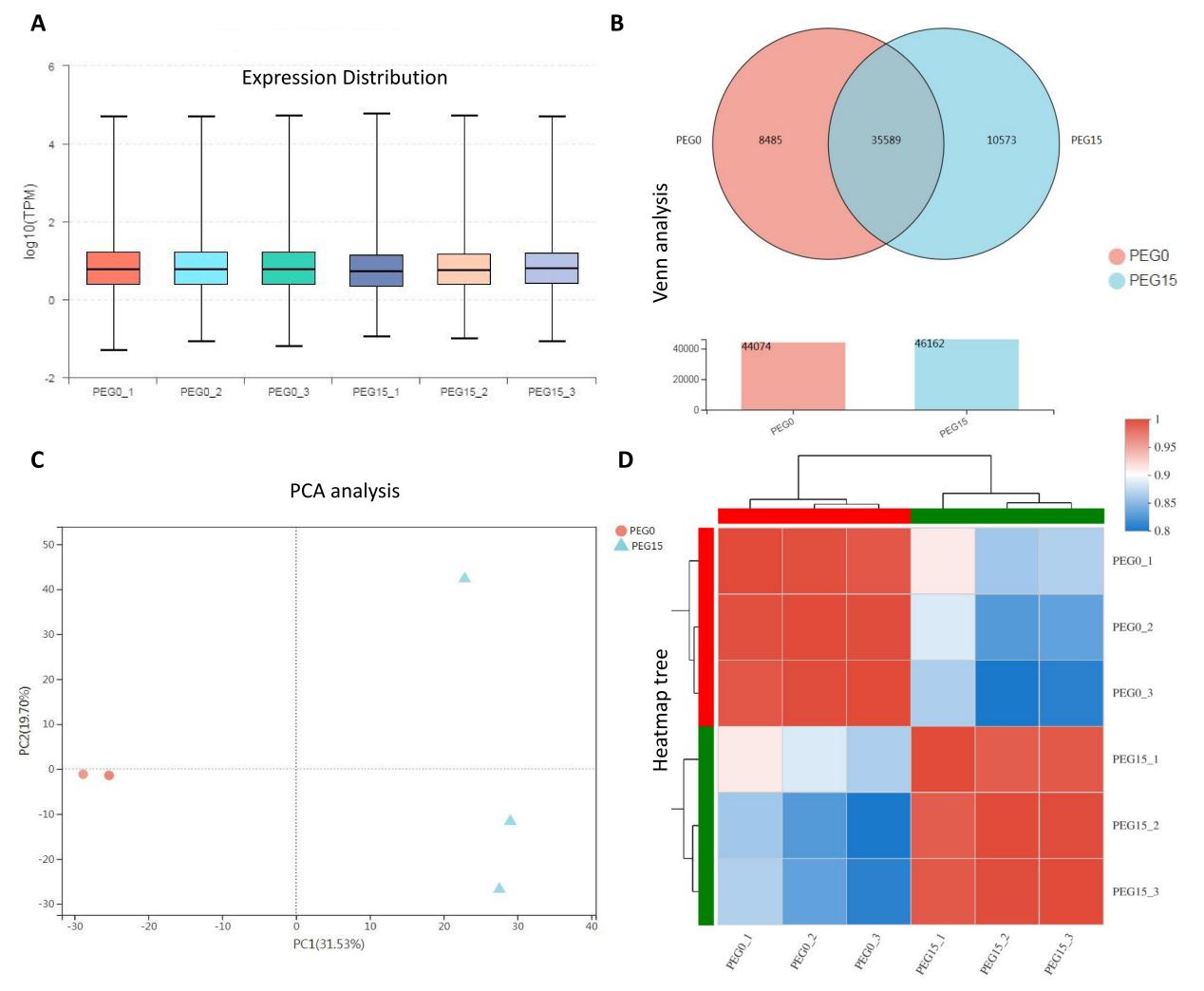


FIGURE S4 Analysis of RNA sequencing data of six samples under normal and 15% PEG6000 conditions. (A) The expression distribution analysis; (B) Venn analysis; (C) PCA analysis of six samples; (D) Correlation analysis of six samples.
